# Supplementary figures and images for: Diversified caching algorithm with cooperation between edge servers
Source: PeerJ Comput Sci. 2025 Apr 30;11:e2824. doi: 10.7717/peerj-cs.2824 (PMC12190530; doi:10.7717/peerj-cs.2824)

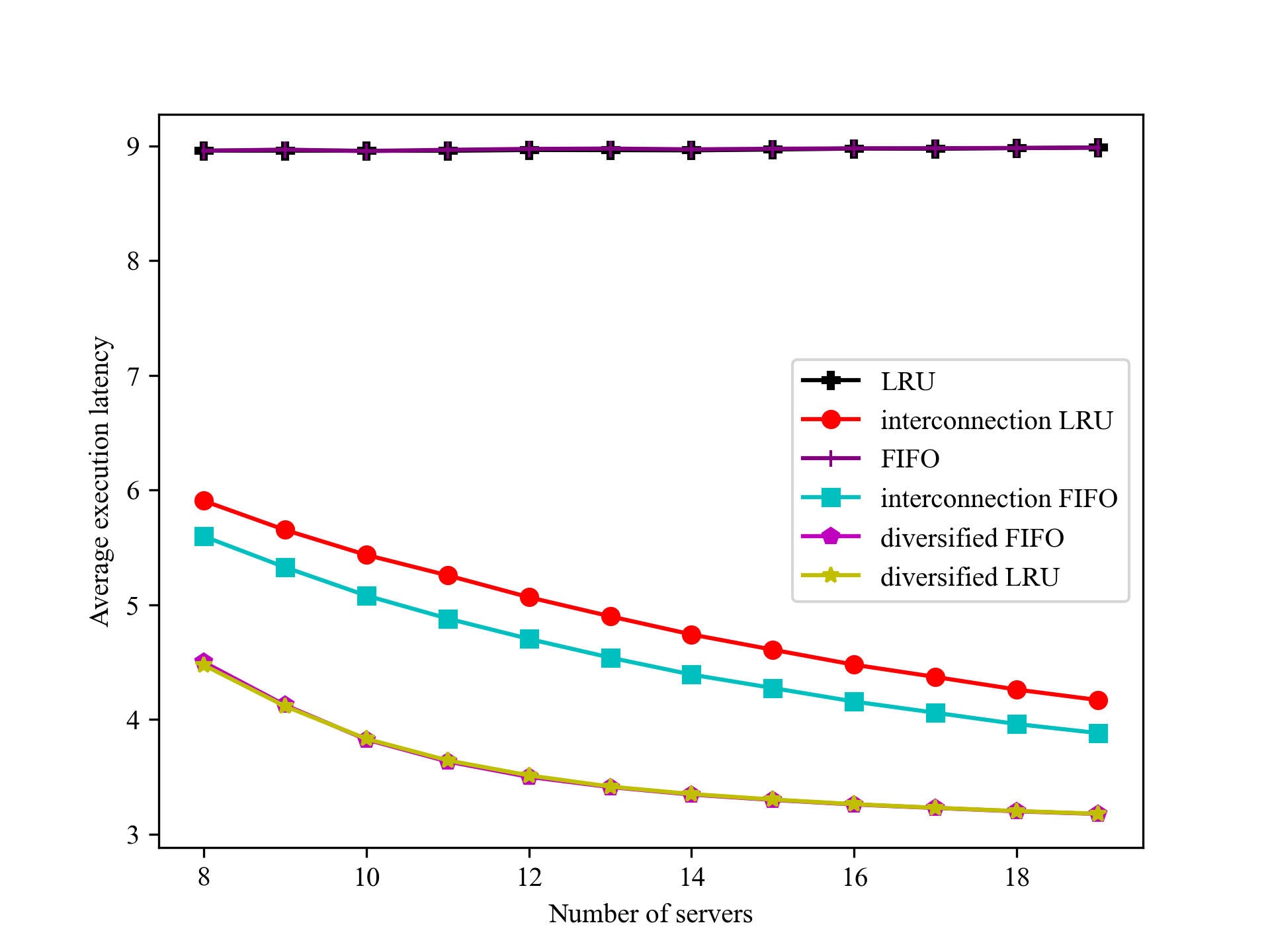

Supplement: Supplemental Information 2 [file peerj-cs-11-2824-s002.zip › program/datas/Average execution latency.png]

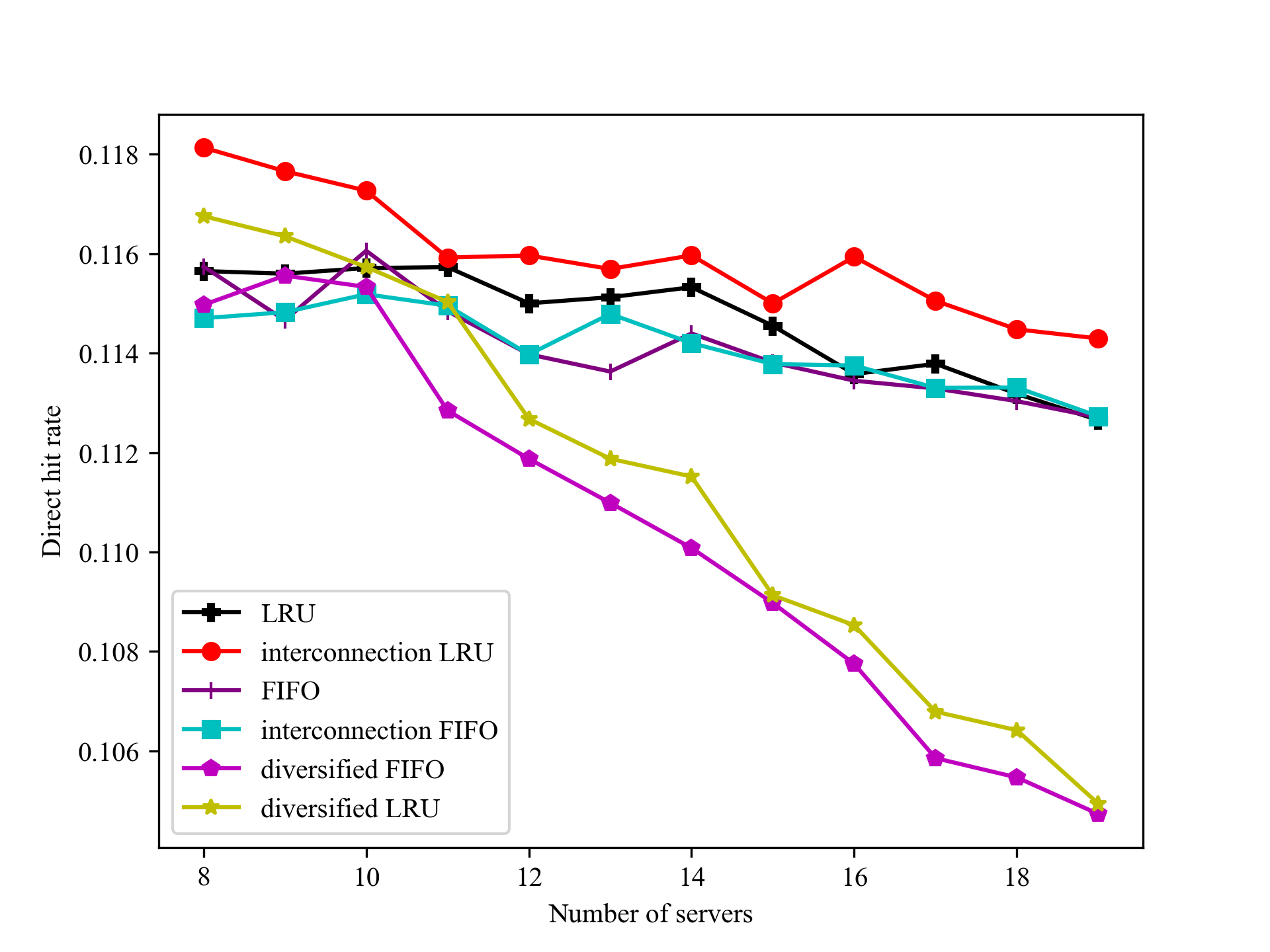

Supplement: Supplemental Information 2 [file peerj-cs-11-2824-s002.zip › program/datas/Direct hit rate.png]

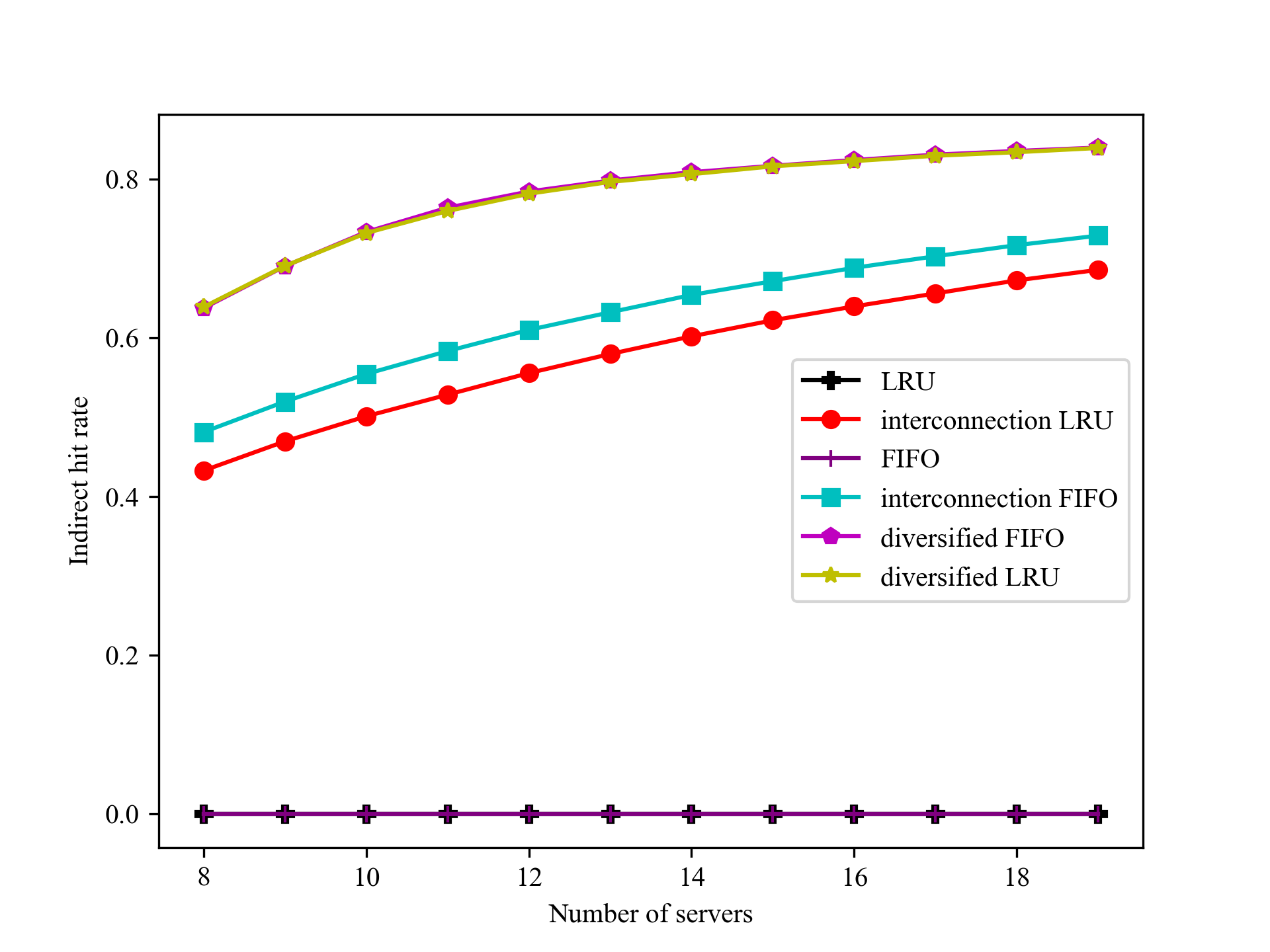

Supplement: Supplemental Information 2 [file peerj-cs-11-2824-s002.zip › program/datas/Indirect hit rate.png]

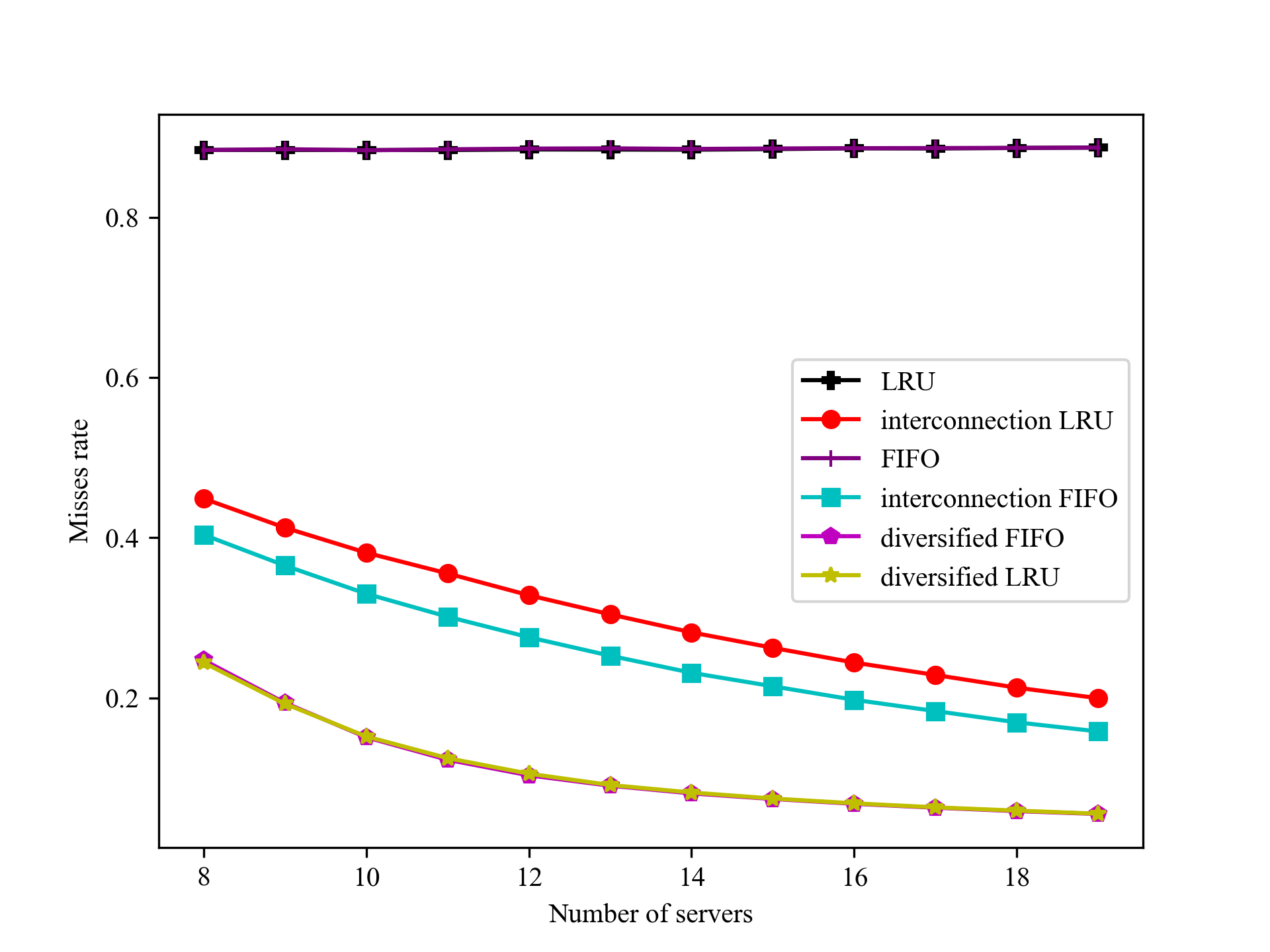

Supplement: Supplemental Information 2 [file peerj-cs-11-2824-s002.zip › program/datas/Misses rate.png]

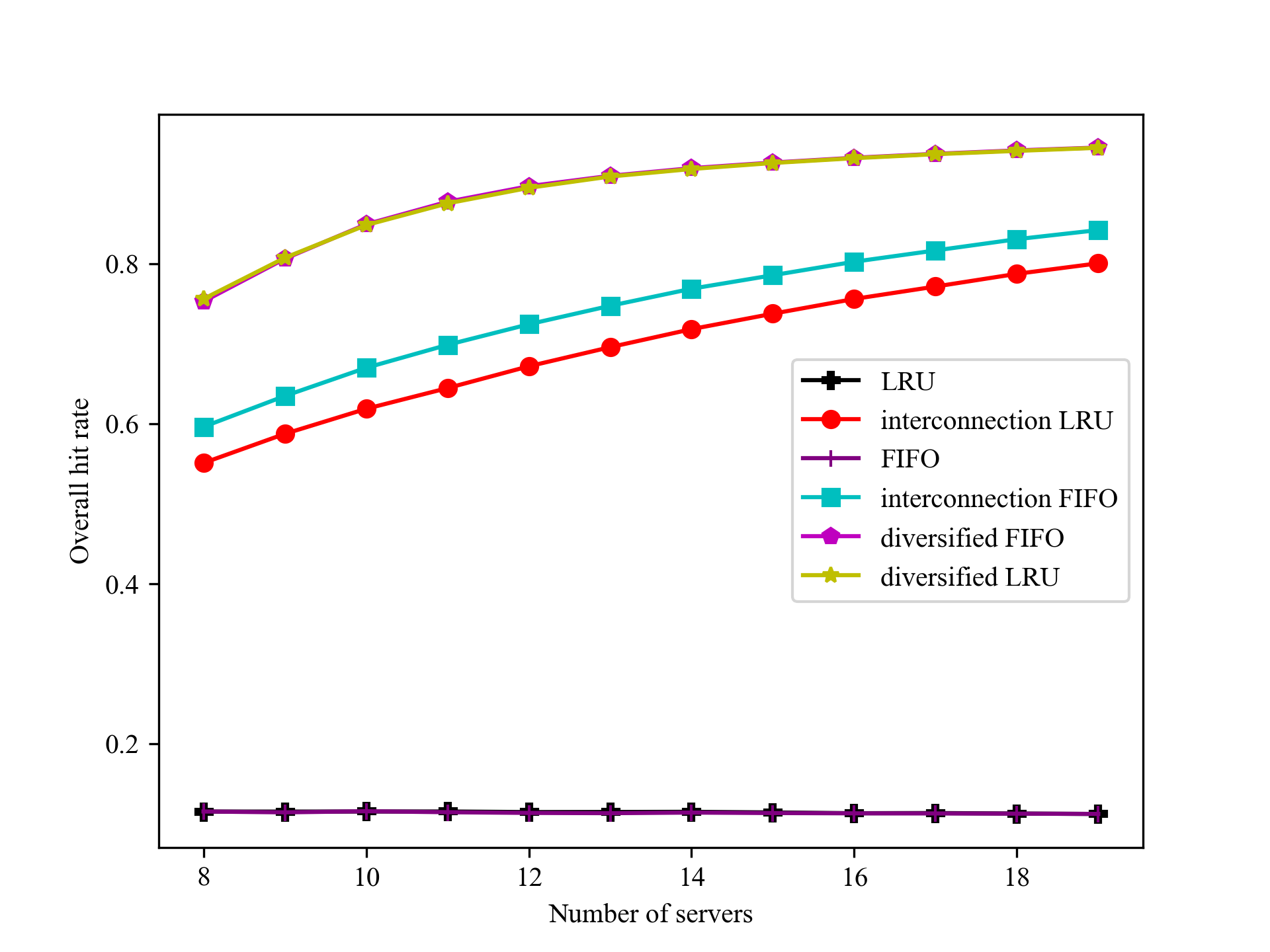

Supplement: Supplemental Information 2 [file peerj-cs-11-2824-s002.zip › program/datas/Overall hit rate.png]
